# Characteristics of Victims of Fall-Related Accidents during Mountain Hiking

**DOI:** 10.3390/ijerph17031115

**Published:** 2020-02-10

**Authors:** Martin Faulhaber, Gerhard Ruedl, Friedemann Schneider, Dagmar Walter, Regina Sterr, Wolfgang Schobersberger, Fabian Schwendinger, Elena Pocecco

**Affiliations:** 1 Department of Sport Science, University of Innsbruck, 6020 Innsbruck, Austria; gerhard.ruedl@uibk.ac.at (G.R.); schwendinger.fabian@web.de (F.S.); elena.pocecco@uibk.ac.at (E.P.); 2 Austrian Society of Alpine and High Altitude Medicine, 6414 Mieming, Austria; wolfgang.schobersberger@tirol-kliniken.at; 3 Department for Trauma Surgery and Sports Medicine, Medical University of Innsbruck, 6020 Innsbruck, Austria; friedemann.schneider@tirol-kliniken.at; 4 Austrian Board of Alpine Safety, 6020 Innsbruck, Austria; dagmar.walter@alpinesicherheit.at (D.W.); regina.sterr@alpinesicherheit.at (R.S.); 5 Institute for Sports Medicine, Alpine Medicine and Health Tourism (ISAG), Tirol Kliniken GmbH, 6020 Innsbruck, Austria; 6 Private University for Health Sciences, Medical Informatics and Technology (UMIT), 6060 Hall/Tyrol, Austria; 7 Pedagogical University Tyrol, 6020 Innsbruck, Austria

**Keywords:** mountain sports, accident, emergency, fall, risk

## Abstract

The study evaluated characteristics of non-fatal mountain hiking accidents caused by falls. Questionnaires were sent to mountain hikers who suffered a fall-related accident in Tyrol (Austria) during a 3-year period. The questionnaire included details of socio-demographic data, physical activity, medication intake, defective vision, breaks, fluid intake, level of fatigue, muscle soreness, use of backpacks, use of hiking sticks, and type of shoes. Data of 405 individuals (57% females and 43% males) were included in the analyses. Victims were 56 ± 15 years of age, had a body mass index of 24.8 ± 3.5, and indicated 4.2 ± 3.9 h/week regular physical activity. A defective vision was reported by 70% of the victims, breaks were frequent (in 80%), and alcohol intake was rare (4%) among the interviewed hikers. Subjective level of fatigue was low and only 5% reported muscle soreness. A backpack was carried by 83% of the victims and the average weight was higher in males compared to females. The majority (61%) of the victims wore ankle-height hiking shoes with a profiled sole. Victims of non-fatal falls in mountain hiking are older than the general population of mountain hikers and are often afflicted with defective vision.

## 1. Introduction

Mountain-sport activities are practiced by an increasing number of persons. In 2001, the number of tourists visiting altitudes above 2000 m was estimated at 40 million people per year in the Alps and worldwide, an annual sum of 100 million high-altitude tourists was assumed [1]. In the Alps, mountain hiking is the most attractive mountain-sport activity during the summer season with many millions of hikers in Austria each year [2] including healthy people, but also individuals with pre-existing chronic diseases [3].

Mountain hiking is recommended as a suitable physical activity for health prevention in young and elderly persons [4] due to it requiring use of a large muscle mass for a prolonged period of time at a predominantly moderate intensity [5]. In addition to the physical activity of mountain hiking itself, the exposure to moderate altitudes and therefore to mild hypoxic conditions, may have additional health benefits [6].

The other side of the undoubtedly positive health effects of mountain hiking activities is the risk of accidents, emergencies, and even fatalities. Previous studies calculated a mortality rate of about 4 deaths per 100,000 hikers per year [2], which is about 4-fold compared to alpine skiing [7]. Falls are responsible for nearly 50% of all fatal and non-fatal accidents during mountain hiking [8,9]. Therefore, the prevention of falls could effectively contribute to reduce accidents, to improve safety, and to optimize the benefit-risk-ratio in mountain hiking. Present research in mountain hiking focused mainly on cardiovascular emergencies. Several risk factors for cardiovascular emergencies (i.e., sudden cardiac deaths) during mountain hiking were identified and general preventive measures have been developed and published [2,8]. However, despite the high frequency, systematic research on mountain hiking accidents caused by falls is scarce and studies dealing with falls not related to mountain sports [10,11] cannot be used to derive recommendations for mountain hiking since external (e.g., terrain) and internal (e.g., fitness of the persons) circumstances are completely different in mountain hiking. Data of previous hiking-specific studies rely on routine data collection of rescue organizations or police reports [9,12,13] and, therefore, information is limited. Therefore, the goal of the present study was to provide detailed characteristics of non-fatal mountain hiking accidents caused by falls.

## 2. Materials and Methods

### 2.1. General Study Design

The present study was a 3-year (2016 to 2018) prospective trial focusing on non-fatal accidents caused by falls during mountain hiking in Tyrol (Austria). All subjects gave their informed consent for inclusion before they participated in the study. The study was conducted in accordance with the Declaration of Helsinki 2013 and the protocol was approved by the Board for Ethical Questions of the University of Innsbruck (certificate of good standing 07/2016).

### 2.2. Selection of Included Accidents and Victims

The Austrian Alpine Police (part of the Ministry of the Interior) routinely documented accidents during mountain-sports activities in the Austrian Alps. During the summer season, this documentation included nearly all accidents and emergencies with an emergency call via the emergency call center or the local mountain rescue service and all fatalities that occurred in the Austrian mountains. Instructed staff of the Austrian Alpine Police was responsible for data input using standardized forms and the database was updated daily. The standardized forms include an identification number, the type of the mountain-sport activity, the location of the accident (e.g., federal province), a rough classification of the accident (e.g., fall, cardiovascular emergency, exhaustion etc.), demographic aspects of the victims, and a short description of the accident’s sequence of events. Names and addresses of the victims are not part of the database and remain in the documents of the Austrian Alpine Police to guarantee the anonymity of victims. This database provided the primary source for the selection of the accidents relevant for this study.

The Austrian Board of Alpine Safety had access to the database of the Austrian Alpine Police for regular national accident reports. For the present study, the Austrian Board of Alpine Safety selected accidents, which fulfilled the following criteria: (1) non-fatal, (2) located in the federal province Tyrol (without east Tyrol), (3) categorized to the activity ‘hiking/mountaineering’, and (4) categorized to the type ‘fall’ during the summer seasons (May 1st to October 31st) each year. Data of the selected accidents and the victims were extracted from the database and transmitted to the Department of Sport Science (University Innsbruck) each week for further analyses.

In the next step, only victims of full age and with permanent residence in Austria or Germany were included. This procedure was due to organizational reasons of sending prepared questionnaires in the German language including a stamped return envelope to the victims (see below) and should not have resulted in a relevant selection since nearly 90% of the victims in the Austrian Alps are Austrian or German nationality [13]. Additionally, the short description of the accidents sequence of events and the location were checked to decide if the accident happened during mountain hiking or during another activity classified as ‘hiking/mountaineering’ by the Austrian Alpine Police. Thereby, according to the definition of mountain hiking, high-altitude mountaineering (e.g., accidents during a glacier traverse), and other activities (e.g., accidents during hunting or mushroom collecting) were excluded. Subsequently, all cases fulfilling the inclusion criteria were selected and corresponding victims were defined as potential study participants.

**Figure 1:**
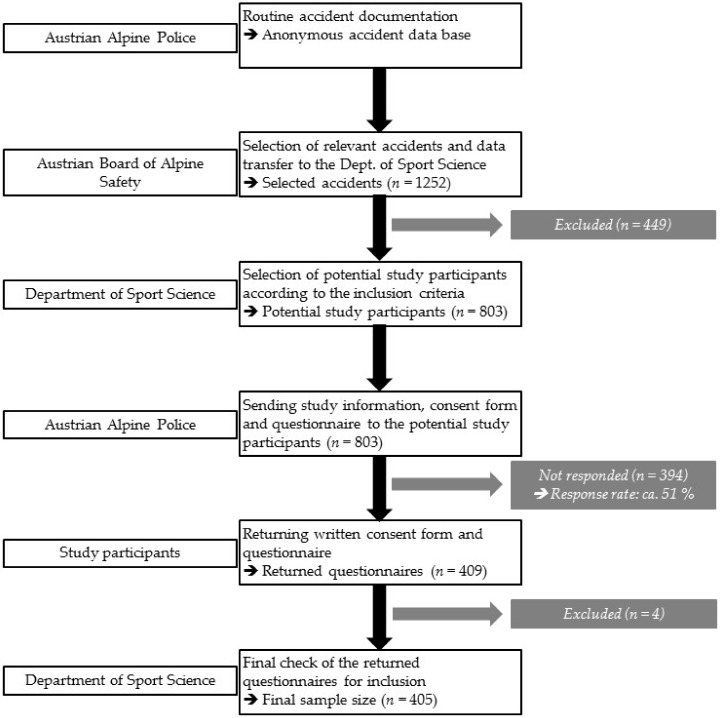
Flow chart showing the procedure of accident inclusion, participants’ response, and selection of the final sample size included into analyses.

On a weekly basis, the Austrian Alpine Police was informed about the identification numbers of the selected cases in order to send the study documents in print form to the victims by mail. The study documents included an information letter about the study goals and the cooperating institutions, a participant information and consent form, and the questionnaire for data acquisition (details see below). The persons were asked to give written informed consent, to fill in the questionnaire, and to send both back to the Department of Sport Science using the stamped return envelope. Only returned questionnaires with the signed consent form were included for subsequent procedures. Returned questionnaires were checked in order to see if the accidents fulfilled the criteria (i.e., mountain hiking as activity and accident primary caused by a fall) for final inclusion into the study. A fall-related accident was accepted when the person slipped, stumbled, or twisted during mountain hiking (e.g., falls during a stay on a mountain hut were not accepted) which consequently resulted in a fall. In case of missing or ambiguous responses in the questionnaire, study participants were contacted by e-mail or phone to complete data from the questionnaire. This procedure was applied and has been proven in previous studies [2]. The flow chart in Figure 1 illustrates the procedure of accident inclusion, participant responses, and selection of the final sample size included into analyses.

### 2.3. Data Collection: Questionnaire

The questionnaire was based on our previous studies [2] and additionally on other studies in mountain sports [14]. Expert interviews were used to construct items that could not be adopted from validated published questionnaires. A pilot project in mountain hikers (*n* = 50) was conducted during the summer season 2015 to validate the questionnaire, to improve clarity, and to facilitate statistical analysis before starting the main evaluation.

The questionnaire included the following details:

1. socio-demographic details (e.g., age, sex, nationality, height, and body weight);
2. extent (hours per week) of regular physical activity;
3. regular medication intake (yes or no);
4. defective vision, e.g., hyperopia (yes or no);
5. breaks (yes or no), fluid intake (l), and alcohol consumption (yes or no) during the tour until the accident happened;
6. level of fatigue at the time of the accident: perceived exertion at the time of the accident on a 0 (no fatigue at all) to 10 (completely exhausted) numeric rating scale;
7. muscle soreness (yes or no);
8. use of backpack (yes or no) and estimated weight (kg);
9. use of hiking sticks (yes or no);
10. type of shoes: (a) running shoes, trainers or sneakers, (b) flat hiking shoes with a profiled sole, (c) ankle-height hiking shoes with a profiled sole, (d) ankle-height mountaineering boots with a rigid sole suitable for use of crampons, (e) other type of shoes;
11. an exact description of the sequence of events during the accident and the circumstances. Based on this information the type of activity (mountain hiking) and the primary cause of the accident (fall) could be verified and falls as a consequence of medical conditions (e.g., myocardial infarction) could be excluded. Additionally, injury locations could be determined.

### 2.4. Statistical Analysis

Statistical analyses were performed using SPSS 24.0 (IBM, Vienna, Austria). Data are presented as means ± standard deviations (SD) or frequencies (95% confidence interval). Differences between subgroups were evaluated by unpaired t-tests (interval-scaled and normally distributed data), Mann–Whitney U-tests (interval-scaled data without normal distribution or ordinal-scaled data), Chi-square exact tests (nominal-scaled data) as appropriate. *p*-values <0.05 (two-tailed) were considered to indicate statistical significance.

## 3. Results

**Figure 2:**
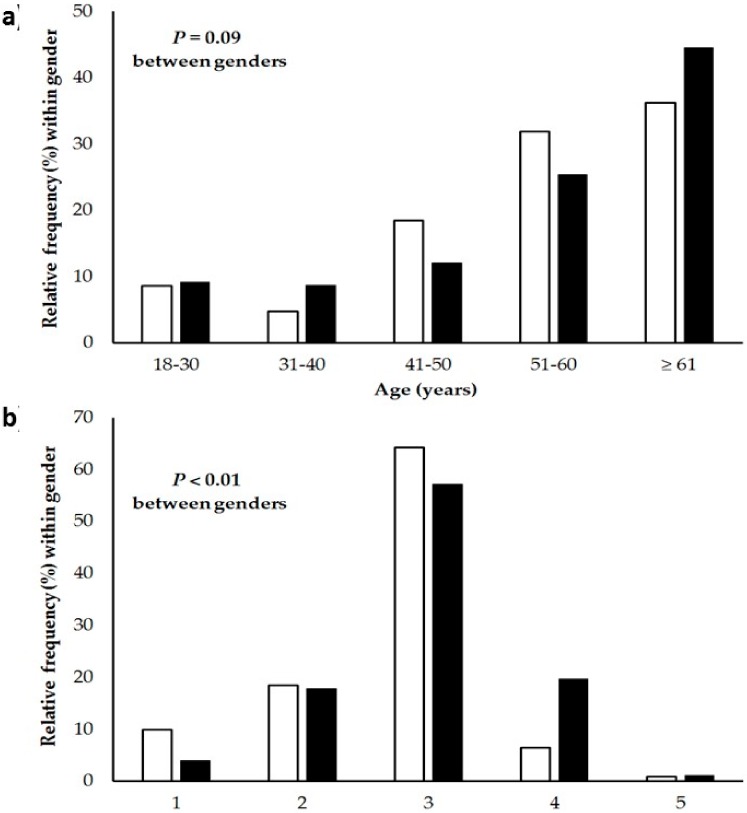
(**a**) Relative frequencies of different age groups within female (white columns) and male (black columns) victims. (**b**) Relative frequencies of different types of shoes within female (white columns) and male (black columns) victims. 1 = running shoes, trainers or sneakers; 2 = flat hiking shoes with a profiled sole; 3 = ankle-height hiking shoes with a profiled sole; 4 = ankle-height mountaineering boots with a rigid sole suitable for use with crampons; 5 = other type of shoes.

Data of 405 victims, 232 (57%) females, and 173 (43%) males, were included in the analyses. They were of German (79%) or Austrian (21%) nationality. Characteristics of the victims and circumstances of the accidents are shown in Table 1. A gender-specific distribution of age groups is shown in Figure 2a. Victims with defective vision were about 7 years older compared to persons without (58 ± 14 versus 51 ± 16 years, *p* < 0.01). Among the persons wearing a backpack, the average backpack weight was 5.6 ± 4.2 kg in females and 7.0 ± 3.8 kg in males (*p* < 0.01 between genders, *n* = 314). However, relative backpack weight (calculated as backpack weight/body weight × 100) did not differ between female (9% ± 7%) and male victims (9% ± 5%; *p* = 0.94 between genders). When focusing on the persons wearing a backpack (*n* = 334), 60% used hiking sticks; backpacks of these individuals were about 40% heavier compared to the individuals not using hiking sticks (7.4 ± 4.0 versus 5.2 ± 3.9 kg, *p* < 0.01). With respect to the whole sample, about 7% wore running shoes, trainers, or sneakers, 18% flat hiking shoes with a profiled sole, 61% ankle-height hiking shoes with a profiled sole, 12% ankle-height mountaineering boots with a rigid sole suitable for use with crampons, and 1% other types of shoes with significant differences between genders (Figure 2b).

In 396 of the 405 accidents, the injury locations could be identified. The ankle joint was the most frequent injury location (42.4%), followed by the head (13.4%), and the lower leg (without ankle) (10.6%). No significant differences in body weight (*p* = 0.10), body mass index (*p* = 0.72), subjective level of fatigue (*p* = 0.11), backpack weight (0.10), and the type of shoes (0.84) were observed when comparing the circumstances of accidents with ankle injuries with the circumstances of the other accidents. However, the proportion of female hikers was significantly higher in the accidents with ankle injuries compared to the other accidents (72.6 versus 46.1%, *p* < 0.01).

## 4. Discussion

The present study showed a high percentage of elderly persons among hikers sustaining a fall during mountain hiking requiring an emergency call. Additionally, a high percentage of the victims suffered from a visual impairment. We detected slight differences between female and male victims in body mass index (BMI), regular physical activity, the absolute weight of backpacks, and the type of shoes. To the best of our knowledge, this is the first study adding epidemiological data on persons who had an accident caused by falling during mountain hiking beyond routine data of rescue organizations or insurances.

The relatively high age of the victims in this study varies markedly from the characteristics of a general population of mountain hikers in the Austrian Alps. Faulhaber et al. reported a mean age of about 42 years [3], which is about 14 years lower compared to victims in this study indicating that a higher age seems to increase the risk for fall-related accidents in mountain hiking. It has to be mentioned that this observation was shown in various other areas of life [15] and in addition, is not specific for falls during mountain hiking since Burtscher et al. showed an age-dependent sudden cardiac death risk in mountain hiking [16].

The fact that 70% of the victims were afflicted by a defective vision might be partly due to the relatively high mean age, with nearly 70% proportion of individuals being above 50 years among the victims. Nevertheless, data from daily life show that the risk of falls is associated with the presence of visual impairment within a population of elderly persons (age above 50 years) [17].

BMI of male victims was higher than females’ one (25.7 vs. 24.1) and more men (52%) than women (34%) were overweight (BMI > 25). These results are in line with the results of a cohort study on 184,697 Austrian adults (85,000 men, 99,697 women), which showed higher percentages of women in comparison to men in the BMI-classes below 22.4 (42.4% vs. 21.3%, respectively) [18]. Other studies, conducted on German and European populations, confirm a higher mean BMI of adult men compared to women (Germans: 26.1 vs. 25.2; Europeans: 27.1 vs. 25.3) [19,20]. In comparison to mountain hikers in general [3], the victims in the present study had a slightly higher mean BMI. Since an association between an increased BMI and dynamic walking stability was shown in elderly persons [21], the higher BMI in the population of hikers with falls seems to indicate the impact of BMI on the fall-related accident risk during mountain hiking. 

Interviewed male hikers practiced a higher mean amount of physical activity compared to women (4.7 vs. 3.9 h per week) but a similar percentage (38% of the female and 35% of the male victims) did not reach the level of regular physical activity of 150 min per week recommended by the WHO [22]. The interviewed hikers can be assumed as above-average in terms of physical activity level compared with the general population, e.g., in Germany, with 65% of the females and 56% of the males below the WHO recommendation [23]. However, the victims of falls in this study seem to be less physically active compared with a general population of mountain hikers interviewed in a survey in the Austrian Alps [3]. The observed differences in regular physical activity compared to the general population of mountain hikers supports the speculation that a low amount of regular physical activity at least partly contributes to a higher risk of fall-related accidents during mountain hiking. 

The absolute weight of backpacks was higher in men compared to women; however, adjusted to body weight, this difference disappeared. It has been reported that loaded walking alters the gait of older adults of both sexes and that unstable loads reduce dynamic stability compared to unloaded walking [24]. Thereby, it can be assumed that heavy and/or poorly balanced backpacks, e.g., heavy loads in the upper part of the backpack, or loose or unfixed equipment hanging outside the backpack, can increase the risk of falls during mountain hiking. Since nearly 80% of the female and nearly 90% of the male victims had a backpack, this aspect should be addressed in preventive measures.

The analysis of the type of shoes showed that most of the mountain hikers wore specific ankle-high boots for hiking or mountaineering (about 71% of the females and about 77% of the males). This indicates that most of the victims had a fall despite use of appropriate footwear. This percentage of accident victims amounted to nearly 90% (for both genders) when flat hiking boots, equipped with a profiled sole comparable to mountaineering boots, were additionally assumed as adequate footwear. The significant difference between genders was mainly caused by the higher percentage of running shoes, trainers, or sneakers in females compared to males (9.9% vs. 4.0%). If this difference is caused by the selected hiking terrain, e.g., female hikers more often select easy terrain compared to male hikers, or by other factors, cannot be clarified by the present data. 

The present study has several limitations. First, the primary data source were accidents with an emergency call and a routine documentation by the Austrian Alpine Police. Therefore, accidents without a professional rescue (e.g., rescue by other hikers or transport by private car to a physician or a hospital) were not taken into account. Unfortunately, the number of these lost cases cannot be estimated with sufficient accuracy. Furthermore, only Austrian and German victims were included into the study and hikers of other nationalities might differ in their characteristics (e.g., equipment). In combination with the response rate of about 50%, both factors could have resulted in a selection bias. Second, the circumstances of the accident were recorded retrospectively. Although this procedure seems to be the only practicable way, participants may have found it difficult to correctly answer some of the questions resulting in a potential information bias. This seems to be the case for questions related to very transient factors, such as the subjective level of fatigue or muscle soreness, while it is relatively unlikely for stable factors (e.g., the type of shoes). Therefore, results of subjective ratings should be interpreted with caution. Third, this study presents circumstances of the accidents and characteristics of the victims and thereby provides important information. However, it does not classify these factors as risk or protective factors as only injured persons were enrolled and the denominators of hiking accidents resulting in falls remain unknown. Therefore, future studies should be designed as comparisons with hikers without accidents to identify potential risk factors for falls during mountain hiking and should include objective evaluations of the resulting injuries.

## 5. Conclusions

Victims of non-fatal falls in mountain hiking are markedly older than the general population of mountain hikers and are often afflicted by visual impairments. Therefore, defective vision, eventually in combination with or without the use of a visual aid, could play a major role in falls during mountain hiking. Therefore, mountain hikers should regularly check their visus and if necessary, the degree of their visual aid. Hikers with defective vision should take care, especially during downhill walking. The present data did not identify a special type of boot that is frequently worn by victims of falls and therefore, the potential importance of the type and the characteristics of shoes remains unclear. Further evaluations should focus on the identification of risk factors to derive evidence-based recommendations for the prevention of falls in mountain hikers.

## Figures and Tables

**Table 1:** Characteristic of the victims and circumstances of the accident.

Characteristics	Total	Females	Males	*p*-Value
**Age (years)**	56 ± 15	56 ± 14	57 ± 16	0.34
**Height (cm)**	171 ± 8	166 ± 6	178 ± 6	<0.01
**Body weight (kg)**	73 ± 13	67 ± 11	81 ± 11	<0.01
**BMI**	24.8 ± 3.5	24.1 ± 3.6	25.7 ± 3.1	<0.01
**BMI > 25 (*n*, %)**	169 (42)	79 (34)	90 (52)	<0.01
**Regular physical activity (h/week)**	4.2 ± 3.9	3.9 ± 3.2	4.7 ± 4.6	0.03
**Regular physical activity** ** <150 min/week (*n*, %)**	147 (36)	87 (38)	60 (35)	0.56
**Regular medication (*n*, %)**	115 (39)	86 (37)	69 (40)	0.55
**Defective vision (*n*, %)**	279 (70)	164 (71)	115 (68)	0.47
One or more breaks * (*n*, %)	318 (80)	181 (80)	137 (80)	0.93
Fluid intake * (*l*)	1.0 ± 0.7	1.0 ± 0.7	1.0 ± 0.7	0.38
Alcohol consumption * (*n*, %)	20 (4)	9 (4)	11 (6)	0.26
Subjective level of fatigue **	2.4 ± 2.3	2.4 ± 2.3	2.5 ± 2.2	0.70
**Muscle soreness (*n*, %)**	19 (5)	9 (4)	10 (6)	0.37
**Backpack (*n*, %)**	334 (83)	184 (79)	150 (87)	0.05
**Use of hiking sticks (*n*, %)**	233 (58)	129 (56)	104 (60)	0.36

Values are means ± SD or absolute (relative) frequencies for the total sample and separated for female and male victims. Sample size varies due to incomplete data (*n* = 391–405 for the total sample size, *n* = 224–232 for females, *n* = 167–173 for males). *p*-value refers to differences between female and male victims. * Breaks/fluid intake/alcohol consumption during the tour at which the accident happened. ** Subjective level of fatigue at the time the accident happened.
